# New developments and opportunities in drugs being trialed for amyotrophic lateral sclerosis from 2020 to 2022

**DOI:** 10.3389/fphar.2022.1054006

**Published:** 2022-11-28

**Authors:** JingSi Jiang, Yan Wang, Min Deng

**Affiliations:** Institute of Medical Innovation and Research, Peking University Third Hospital, Beijing, China

**Keywords:** amyotrophic lateral sclerosis, amyotrophic lateral sclerosis∗/drug therapy, amyotrophic lateral sclerosis∗/genetics, clinical trials as topic, drug development

## Abstract

Amyotrophic lateral sclerosis (ALS) is a progressive neurodegenerative disorder that primarily affects motor neurons in the brain and spinal cord. In the recent past, there have been just two drugs approved for treatment, riluzole and edaravone, which only prolong survival by a few months. However, there are many novel experimental drugs in development. In this review, we summarize 53 new drugs that have been evaluated in clinical trials from 2020 to 2022, which we have classified into eight mechanistic groups (anti-apoptotic, anti-inflammatory, anti-excitotoxicity, regulated integrated stress response, neurotrophic factors and neuroprotection, anti-aggregation, gene therapy and other). Six were tested in phase 1 studies, 31 were in phase 2 studies, three failed in phase 3 studies and stopped further development, and the remaining 13 drugs were being tested in phase 3 studies, including methylcobalamin, masitinib, MN-166, verdiperstat, memantine, AMX0035, trazodone, CNM-Au8, pridopidine, SLS-005, IONN363, tofersen, and reldesemtiv. Among them, five drugs, including methylcobalamin, masitinib, AMX0035, CNM-Au8, and tofersen, have shown potent therapeutic effects in clinical trials. Recently, AMX0035 has been the third medicine approved by the FDA for the treatment of ALS after riluzole and edaravone.

## Introduction

Amyotrophic lateral sclerosis (ALS) is a progressive neurodegenerative disorder that leads to the loss of motor neurons in the brain and spinal cord, causing loss of muscle control. It begins with weakness and muscle twitches and spreads to involve most muscles. Most patients die in 3–5 years after symptom onset because of respiratory paralysis (Brown and Al-Chalabi, 2017).

Most cases of ALS do not have a known cause, and only 5%–10% of cases are inherited with mutations in genes such as SOD1, FUS, C9ORF72, ATAXIN-2, CCNF, GLT8D1, KIF5A, NEK1, C21orf2, DNAJC7, ANXA11 and TIA (Laffita-Mesa et al., 2013; Brown and Al-Chalabi, 2017). The possible pathological mechanisms of ALS include abnormal accumulation of misfolded or aggregated proteins, oxidative stress, excitotoxicity, mitochondrial dysfunction, endoplasmic reticulum stress and inflammation. These can lead to the loss of neurons and are potential therapeutic targets for ALS (Brown and Al-Chalabi, 2017). In addition, denervation leads to degeneration and atrophy of skeletal muscle, which may also play a role in the etiopathogenesis of ALS through changes in sarcolemma ion channels, mitochondrial dysfunction, muscle metabolism, muscle regeneration, and RNA processing (Manzano et al., 2021; Tarantino et al., 2022).

Currently, there is no cure for ALS and few treatments available, none of which have a profound effect on survival. In the recent past, only riluzole and edaravone have been approved by the U.S. Food and Drug Administration (FDA) for the treatment of ALS. However, they can only slow the progression for a few months and cannot reverse damage to motor neurons (Jaiswal, 2019). There are many new experimental therapies in development that show promise in treating ALS. Some of these pipeline agents have reported to slow the progression more effectively than riluzole and edaravone. One of them, AMX0035, has been approved by the FDA on September 2022, although the evidence is limited.

In this review, we will discuss the three FDA-approved drugs and summarize 53 new drugs that have been evaluated in clinical trials from 2020 to 2022. These are categorized into eight mechanistic groups: anti-apoptotic, anti-inflammatory, anti-excitotoxicity, regulated integrated stress response (ISR), neurotrophic factors and neuroprotection, anti-aggregation, gene therapy and other mechanisms. We will also highlight the most promising new treatments for ALS.

## Drugs

### Approved drugs

Current treatment for ALS is limited. In the recent past, there has been only two FDA-approved drugs (riluzole and edaravone), which can only extend life by a few months (Brown and Al-Chalabi, 2017). On 29 September 2022, AMX0035 was approved by the FDA for the treatment of ALS. The details of AMX0035 are discussed in Section 2.2.1.

#### Riluzole

Riluzole is an anti-glutamate agent developed by Sanofi and was the first medicine approved for the treatment of ALS by the FDA in 1995. It can inhibit the overexcitation of motor neurons and decrease excitotoxic neuronal cell death by inhibiting glutamate presynaptic release and protein kinase C, inactivating voltage-dependent sodium channels (Benoit and Escande, 1991), and inactivating potassium channels slower (Xu et al., 2001). The main adverse events reported are nausea, weakness and transiently elevated liver enzyme levels (Jaiswal, 2019).

Riluzole can slow the progression of ALS, extend survival (Bensimon et al., 1994) and delay the time to tracheostomy (Lacomblez et al., 1996). It also prolonged survival of the last clinical stage of ALS (Dharmadasa and Kiernan, 2018; Fang et al., 2018). However, the benefit is limited. It can only extend the average survival time by 3 months (Bensimon et al., 1994) and cannot reverse the damage to motor neurons.

#### Edaravone

Edaravone is a free radical scavenger that can reduce oxidative stress. It was first found to be effective in Japan. A Japanese-cohort, phase 3, randomized, double-blind, parallel-group clinical trial showed that short-term treatment with edaravone can slow the progression of ALS. The decrease in the Revised ALS Functional Rating Scale (ALSFRS-R) score was lower in the edaravone-treated group than in the placebo group (95% CI 0.99–3.98; *p* = 0.0013) (Writing Group, 2017).

However, the effect of edaravone is still inconclusive and inconsistent, as some trials have reported contradictory results. A phase 3 trial in Japan showed that edaravone slowed the decline in the ALSFRS-R score (Writing Group, 2017), and Brookes et al. found that edaravone prolonged survival for 6 months in America (Brooks et al., 2022). However, Witzel et al. found that edaravone did not provide a clinically relevant benefit on the progression rate, time to ventilation or survival in Germany (Witzel et al., 2022). Similar results were shown in Italy (Lunetta et al., 2020). Due to the lack of effectiveness, edaravone is not approved in Europe.

The disparities in outcomes may be attributed to genetics, as there are ethnic differences in the response of the medicine (Jayasinghe et al., 2022). The studies show a statistically significant treatment effect in some Southeast Asian countries (Japan (Writing Group, 2017) and Korea (Park et al., 2020)) and America (Brooks et al., 2022), but there is no significant improvement in the outcome in European (Germany (Witzel et al., 2022) and Italy (Lunetta et al., 2020)), Indian (Tomar et al., 2022), and some Middle Eastern countries (Kuwait (IsmailMassoud et al., 2021) and Iranian (Eishi-Oskouei and Basiri, 2021)) cohorts.

### New drugs in clinical trials

Fifty-two new drugs are discussed in this part, divided into eight mechanistic groups. The details of the newest clinical trials of each drug are listed in Table 1.

**TABLE 1:** The mechanism and clinical trials details of 53 new drugs under investigation for the treatment of ALS. Including the phase, countries, status, number of patients, start and (estimated) completion year, registration number, primary and secondary endpoints, the results and reference.

Candidate	Mechanism	Phase	Status	Country	Patients	Start year	Completion year	Registration number	Primary endpoints	Secondary endpoints	Results	Reference
Anti-apoptotic agents												
AMX0035	reduce ER stress and mitigate mitochondrial dysfunction	Ⅲ	ongoing	United States, France, Germany etc.	600	2021	2024	NCT05021536	Safety, Tolerability, and ALSFRS-R score	SVC, ALSAQ-40,King’s and MiToS Stages, Ventilation Free Survival, EQ-5D descriptive system and the EQ visual analogue scale [EQ VAS],Long-Term Survival	Ongoing	Amylyx, (2021)
Methylcobalamin	eliminate neurotoxic homocysteine	Ⅲ	complete	Japan	130	2017	2020	NCT03548311	ALSFRS-R score	Survival, FVC, homocystein, MMT, Norris scale, Grip Power, ALSAQ-40	Slow the change of ALSFRS-R	Oki et al. (2022)
SAR443820	RIPK1 inhibitor	Ⅱ	ongoing	Canada, France, Germany, China, United States	261	2022	2025	NCT05237284	ALSFRS-R score and CAFS score	SVC, HHD, ALSAQ5, Nfl, TEAE,SAE, PK, survival time	Ongoing	Sanofi, (2022)
GDC-0134	DLK inhibitor	Ⅰ	complete	Canada, Netherlands, United States	54	2016	2020	NCT02655614	Safety and Tolerability	PK and PD	Not safe	Katz et al. (2022)
Anti-inflammatory Drugs												
Masitinib	selective tyrosine kinase inhibitor	Ⅲ	ongoing	United States, Denmark, France, Germany	495	2021	2023	NCT03127267	ALSFRS-R score	ALSAQ-40, CAFS, FVS, HHD, PFS	Ongoing	AB Science, (2017)
NP001	a regulator of macrophage activation	Ⅱb	complete	United States, Canada	138	2016	2017	NCT02794857	ALSFRS-R score	SVC, Time to tracheotomy, and blood inflammatory biomarkers	Failed, but it slowed the decline of ALSFRS-R score and vital capacity of 40–65 subgroup	Miller et al. (2022a)
Verdiperstat	MPO inhibitor	Ⅱ/Ⅲ	ongoing	United States	167	2020	2023	NCT04436510	ALSFRS-R score	SVC, HHD, survival	Ongoing	Cudkowicz, (2020a)
Tegoprubart (AT-1501)	CD40L antibody	Ⅱa	complete	United States, Canada	54	2020	2022	NCT04322149	Safety and Tolerability	--	It is safe and well tolerated and can reduce inflammatory biomarkers	(Anelixis Therapeutics, 2020), (Eledon Pharmaceuticals, 2022)
BLZ945	CSF-1R inhibitor	Ⅱ	ongoing	United States, Finland, Sweden	46	2019	2024	NCT04066244	volume of distribution fot [11C]-PBR28 PET scan	PK	Ongoing	Novartis Pharmaceuticals, (2019)
Proleukin (Aldesleukin)	Enhance Treg function	Ⅱ	complete	France and United Kingdom	304	2017	2021	NCT03039673	Time to death	--	The results have not been posted, as of 2022.08.02	Centre Hospitalier Universitaire de Nīmes, (2017)
Dimethyl fumarate	increase regulatory T-cells	Ⅱ	complete	Australia	107	2018	2021	ACTRN12618000534280	ALSFRS-R score	survival, lower motor neuron function, respiratory function, quality of life, safety and tolerablity	Failed	Vucic et al. (2021a)
Pegcetacoplan	C3 inhibitor	Ⅱ	ongoing	Japan, United States, France, Germany, Italy	249	2020	2024	NCT04579666	CAFS score	TEAE, ALSFRS-R score, SVC, time to death, permanent tracheostomy, or permanent assisted, muslce strength, C-SSRS score	Ongoing	Apellis Pharmaceuticals, (2020)
ANX005	C1q antibody	Ⅱa	ongoing	United States, Canada	24	2021	2023	NCT04569435	TEAE	PK and PD	Ongoing	Annexon, (2021)
Zilucoplan	C5 inhibitor	Ⅱ/Ⅲ	stopped	United States	162	2020	2022	NCT04436497	ALSFRS-R score	SVC, HHD, survival	Failed	(Figueiredo)
Ultomiris (ravulizumab)	C5 inhibitor	Ⅲ	stopped	United States, Denmark, France, etc.	382	2020	2021	NCT04248465	ALSFRS-R score	SVC, Time To Ventilator Assistance-free Survival, TEAE,HDD,Nfl	Failed	AstraZeneca, (2021)
MN-166 (ibudilast)	Inhibit PDE4 and MIF	Ⅱb/Ⅲ	ongoing	United States, Canada	230	2020	2024	NCT04057898	ALSFRS-R score	HHD, ALSAQ-5, Time to survival, TEAE, and Laboratory Values	Ongoing	Oskarsson et al. (2021a)
Tocilizumab	IL-6R inhibitor	Ⅱ	complete	United States	22	2015	2018	NCT02469896	Safety and Tolerability	SVC, ALSFRS-R, HHD, Peripheral Blood Mononuclear Cell (PBMC) Gene Expression, Cytokine Levels in the Plasma, Cytokines and Soluble Interleukin-6 in csf, Peripheral Benzodiazepine Receptor 28 (PBR28) Positron Emission Tomography (PET)	It is safe and well tolerated and can reduce CRP.	Milligan et al. (2021)
Antiexcitotoxicity agent												
Memantine	NMDA receptor inhibitor	Ⅱ/Ⅲ	ongoing	United Kingdom	531	2020	2026	NCT04302870	ALSFRS-R score and survival	ECAS, FVC, King’s ALS Clinical stage, HADS, EQ-5D-5L, AE	Ongoing	Wong et al. (2022)
Mexiletine	sodium channel blocker	Ⅱ	complete	United States	20	2016	2018	NCT02781454	Resting Motor Threshold	Short-interval intracortical inhibition, Motor Evoked Potential, Cortical Silent Period, Strength Duration Time Constant, Threshold Electrotonus, Hyperpolarizing Threshold Electrotonus, Superexcitability,Subexcitability, Frequency of Muscle Cramps	It reduced motor neuron excitability	Adiao et al. (2020)
Ezogabine	KCNQ channel	Ⅱ	complete	United States	65	2015	2018	NCT02450552	Short-interval Intracortical Inhibition	Resting Motor Evoked Potential (MEP) Threshold, Motor Evoked Potential, Cortical Silent Period, Intracortical Facilitation, Electrotonus, Strength Duration Time Constant, Recovery Cycle, Muscle Cramping Frequency, hhd, Proportion of Days With Fasciculations, tolerability	It reduced motor neuron excitability	Wainger et al. (2021)
Penicillin G/Hydrocortisone	GABA inhibitor	Ⅱ	complete	Netherlands	16	2017	2020	EudraCT number 2017–001983-39	ALSFRS-R score	lung function, muscle strength, plasma creatinine, clinical stage, gastrostomy placement, quality of life and AE	Failed	Van Es et al. (2020)
Perampanel	AMPA receptor inhibitor	Ⅱ	complete	Japan	66	2017	2020	NCT03019419	ALSFRS-R score	Manual Muscle Test, FVC	Failed	Aizawa et al. (2022)
Regulate integrated stress response												
Trazodone	NMDA receptor inhibitor	Ⅱ/Ⅲ	ongoing	United Kingdom	531	2020	2026	NCT04302870	ALSFRS-R score and survival	ECAS, FVC, King’s ALS Clinical stage, HADS, EQ-5D-5L, AE	Ongoing	Wong et al. (2022)
Guanabenz	PPP1R15A/PP1c phosphatase complex inhibitor	Ⅱ	complete	Italy	201	2016	2021	Eudract Number 2014–005367-32	proportion of patients progressing to higher stages of disease within 6 months	CSF biomarker (creatinine, albumin, tau, pNfH, TDP43, cystatin C, fetuin A, transthyretin, and CD14/S100ß and pNfH/C3)	Slowed the progression, but high adverse events	Dalla Bella et al. (2021)
IFB-088(Sephin1)	PPP1R15A/PP1c phosphatase complex inhibitor	Ⅱ		France and Italy	42	—	—	—	—	—	have not been approved yet	InFlectis BioScience, (2022)
ABBV-CLS-7262	eIF2B activitor	Ⅰ	ongoing	United State, Canada	30	2021	2023	NCT04948645	Safety, Tolerability, and PK	Ccsf PK, safe and tolerability	Ongoing	Calico Life Sciences LLC, (2021)
DNL343	eIF2B activitor	Ⅰb	ongoing	United States, Netherlands	30	2021	2023	NCT05006352	TEAE	PK	Ongoing	Denali Therapeutics, (2021)
Neurotrophic factors and neuroprotection												
CNM-Au8	catalyze for energy metabolism	Ⅱ/Ⅲ	ongoing	United States	161	2020	2023	NCT04414345	ALSFRS-R score	SVC, Survival, ALSFRS-R	Ongoing	Cudkowicz, (2020b)
Pridopidine	sigma-1 receptor agonist	Ⅱ/Ⅲ	ongoing	United States	163	2020	2023	NCT04615923	ALSFRS-R score	Bulbar Function and speech measured by ALSFRS-R, SVC,HHD,Survival	Ongoing	Cudkowicz, (2020c)
ILB^®^	neurotrophic and myogenic	Ⅱa	complete	Sweden	13	2018	2019	NCT03613571	Safety, Tolerability, Norris rating scale,FVC, functional rating of autonomous and sensory symptoms, PK and PD	ALSFRS-R score	Safe, well tolerated, and improve the ALSFRS-R score	Logan et al. (2022)
G-CSF	inhibit apoptosis, induce neurogenesis, mobilize hematopoietic stem cells (HSCs) and reduce inflammatory activity	Ⅱ	ongoing	Italy	76	2020	--	Eudract 2014–002228-28	ALSFRS-R score	Scale for Testing Muscle Strength,FVC, McGill QoL Questionnaire, Time to death or tracheostomy, Interruption of treatment,SAE	Ongoing	Salamone et al. (2020)
Fasudil	Inhibit ROCK	Ⅱa	ongoing	France, Germany, Switzerland	120	2019	2023	NCT03792490	safery, tolerability	ALSFRS-R score, ECAS, Motor Unit Number Index,SVC, survival,ALSAQ-5	Ongoing	University Medical Center Goettingen, (2019)
Inosine	endogenous antioxidant	Ⅱ	complete	United States	48	2017	2020	NCT03168711	Safety and tolerablity	--	Tolerated. The serious AE was reported in 28.57% (4/14) of patients receiving Inosine and 11.11% (1/9) of those receiving placebo	Massachusetts General Hospital, (2017)
anti aggregation												
AP-101	SOD1 protein antibody	Ⅱa	Ongoing	United States, Canada, Germany, Korea, Sweden	63	2021	2023	NCT05039099	Safety and tolerablity	PK and PD, Nfl	Ongoing	AL-S Pharma, (2021)
Colchicine	induce the expression of HSPB8	Ⅱ	Ongoing	Italy	54	2019	2022	NCT03693781	ALSFRS-R score	TEAE, Tracheostomy-free survival rate, FVC,ALSAQ-40, enhancement of autophagy, stress granules size, number and composition, insoluble species,modifications on extracellular vesicles secretion in blood and CSF, biomarkers of neurodegeneration, biomarkers of inflammation	Ongoing	Mandrioli et al. (2019)
Arimoclomol	Enhance the expression of heat shock protein	Ⅲ	complete	United States, Canada, Italy, Germany etc.	245	2018	2020	NCT03491462	CAFS score	Time to permanent assisted ventilation (PAV)/tracheostomy/death, ALSFRS-R, SVC	Failed	(KemPharm Denmark A/S, 2018), (Inacio, 2021)
Rapamycin	mTOR inhibitor	Ⅱ	complete	Italy	63	2017	2022	NCT03359538	Tregs	safety, ALSFRS-R score, inflammasome, ALSAQ-40,fvc,Tracheostomy-free survival, blood biomarkers, activation and homing capabilities of different T, B, natural killer (NK) cell subpopulations, S6 ribosomal protein, Rapamycin capacity to pass through blood brain barrier	increased Tregs, decreased inflammasome and improved the quality of life	(Azienda Ospedaliero-Universitaria di Modena, 2017), (ENCALS, 2022)
Monepantel	mTOR inhibitor	Ⅰ	ongoing	Australia	12	2022	2023	NCT04894240	PK and Determination of Phase 2 Dose	peripheral blood mononuclear cell phosphorylated ribosomal protein S6 kinase B1 (RPS6KB1) levels, peripheral blood mononuclear cell phosphorylated eukaryotic initiation factor 4 E binding protein 1 (EIF4EBP1) levels, ALSFRS-R, ECAS,SVC, urinary p75 levels MRI,Nfl	Ongoing	PharmAust Ltd, (2022)
Tamoxifen	Enhance autophagy through both mTOR dependent and independent pathway	Ⅰ/Ⅱ	complete	China	20	2014	2019	NCT02166944	ALSFRS-R score	pulmonary function test	Only shortly slow the decline for 6 months. No difference in 12 months	Chen et al. (2020b)
Bosutinib	Src/c-Abl kinase inhibitor	Ⅰ/Ⅱ	ongoing	Japan	25	2022	--	jRCT2051220002	Safety, ALSFRS-R score	--	Ongoing	Haruhisa, (2022)
SLS-005 (trehalose)	activateg Transcription Factor EB and FOXO1 transcription factor	Ⅱ/Ⅲ	ongoing	United States	160	2022	2023	NCT05136885	ALSFRS-R score	SVC,HHD,Survival	Ongoing	Cudkowicz, (2021)
Withaferin A	NF-κB Inhibition	Ⅱ	ongoing	Canada	75	2021	2022	NCT05031351	satey	Short-interval intracortical inhibition, Resting motor threshold, recovery cycle, strength duration time constant	Ongoing	Sunnybrook Health Sciences Centre, (2021)
3K3A-APC	Activate PAR1, inhibit microglia, and repair BBB	Ⅱ	ongoing	Australia	16	2021	2022	NCT05039268	Safety and Microglial Activation in the Motor Cortex	MRI, Monocyte Activation, Cytokine,Chemokine,Nfl, Soluble CD14, Kynurenine Level	Ongoing	Macquarie University, (2021)
AL-001(Latozinemab)	Increase progranulin levels	Ⅱ	ongoing	United States	45	2021	2023	NCT05053035	safety, tolerability, PK and PD,progranulin	Nfl	Ongoing	Alector, (2021)
Tideglusib	GSK-3β inhibitor	Ⅱ	ongoing	Switzerland	98	2021	2024	NCT05105958	Alanine Aminotransferase	Diarrhea	Ongoing	Geneva University Hospitals, (2021)
Gene therapy												
Tofersen (BIIB067)	SOD1 mRNA antisense	Ⅲ	ongoing	United States, Canada, France, Japan, Korea, etc.	150	2021	2027	NCT04856982	Percentage of Participants with Emergence of Clinically Manifest ALS Within 12 Months	Time to Emergence of Clinically Manifest ALS, ALSFRS-R, SVC, Survival,AE, SAE, Nfl, CSF SOD1	Ongoing	Benatar et al. (2022)
ION363/Jacifusen	FUS antisense	Ⅲ	ongoing	United States, Australia, Belgium, Canada, United Kingdom	77	2021	2025	NCT04768972	ALSFRS-R score, Ventilation Assistance-free survival	ALSSQOL-R, Survival, SVC, HHD, Nfl	Ongoing	Ionis, (2021)
WVE-004	C9ORF72 mRNA antisense	Ⅰ/Ⅱ	ongoing	Australia, Canada, United Kingdom, New Zealand, etc.	42	2021	2023	NCT04931862	AE	poly-GP,Pharmacokinetic	Ongoing, Reduce poly-GP	(Meglio, 2022b), (Wave Life Sciences Ltd, 2021)
BIIB078	C9orf7 mRNA antisense	Ⅰ	complete	United States, Canada, Netherlands, Switzerland, United Kingdom	90	2020	2022	NCT04288856	AE and SAE	PK	Safe, but not meet secondary goals. The development is discontinued	(Biogen, 2020), (Hoffman, 2022)
BIIB105	ataxin-2 antisense	Ⅰ	ongoing	United States, Canada, Netherlands, Italy	70	2020	2026	NCT04494256	AE and SAE	PK and PD	Ongoing	Biogen, (2020)
APB-102	a recombinant AAVrh10 that express anti-SOD1 microRNA	Ⅰ/Ⅱ		—	—	—	—	—	—	—	The details of the trial have not been shared	Pinto, (2021)
Engensis (VM202)	Deliver HGF gene through plasmid	Ⅱa	complete	United States, Korea	14	2021	2022	NCT04632225	safety	ALSFRS-R, HHD, Accurate Test of Limb Isometric Strength,ALSAQ-40, Patient Global Impression of Change,SVC, Time to all-cause mortality, gene expression differences in muscle atrophy biomarkers	Safe and well tolerated	Helixmith, (2022)
Other												
Reldesemtiv	fast skeletal muscle troponin activator	Ⅲ	ongoing	United States, Canada, Australia, Germany, etc.	555	2021	2024	NCT04944784	ALSFRS-R score	FVC,HHD,ALSAQ-40	Ongoing	Cytokinetics, (2021)

Note: ALSFRS-R, Amyotrophic Lateral Sclerosis Functional Rating Scale-Revised scores; ALSAQ-40, 40-item Amyotrophic Lateral Sclerosis Assessment Questionnaire; ALSAQ5, Amyotrophic Lateral Sclerosis Assessment Questionnaire; CAFS, The function and survival score; C-SSRS, Columbia Suicide Severity Rating Scale; ECAS, Edinburgh Cognitive and Behavioural ALS Screen; HADS, hospital anxiety and depression scale; FVC, Forced Vital Capacity; SVC, Slow vital capacity; MMT, Manual Muscle Testing; HHD, Hand-held dynamometry; NfL, Neurofilament light chain; TEAE, Treatmentemergent adverse events; SAE, Serious adverse event; AE, adverse event; PK, Pharmacokinetic; PD, Pharmacodynamics; PFS, Progression free survival; CSF, Cerebrospinal Fluid.

#### Anti-apoptotic

AMX0035 is a combination of sodium phenylbutyrate and taurursodiol (tauroursodeoxycholic acid) and is designed to reduce neuronal death by targeting the mitochondria and endoplasmic reticulum (ER) simultaneously. It is a dual UPR-Bax apoptosis inhibitor (Paganoni et al., 2020). Among the two components, sodium phenylbutyrate can ameliorate ER stress by upregulating the master chaperone regulator DJ-1 (Zhou et al., 2011), while taurursodiol can incorporate into the mitochondrial membrane and prevent bax translocation to increase the apoptotic threshold (Rodrigues et al., 2003).

Data from a phase 2 study indicated that AMX0035 was well tolerated. The main adverse events reported were gastrointestinal (diarrhea, nausea, abdominal discomfort, etc.). After 24 months of treatment, the ALSFRS-R score of the AMX0035 group was 2.92 points greater than that of the placebo group. The effects were evident in rapidly progressive ALS phenotypes. However, other secondary endpoints did not differ between the groups, including isometric muscle strength measured by Accurate Test of Limb Isometric Strength, plasma phosphorylated axonal neurofilament H subunit levels (pNF-H), the slow vital capacity (SVC), and the time to death, tracheostomy, or permanent ventilation (Paganoni et al., 2020). Long-term analysis also showed that continuous early treatment with AMX0035 lowered the death risk by 44% and prolonged tracheostomy/PAV-free survival (Paganoni et al., 2022). Analysis using final overall survival intention-to-treat analysis and the rank-preserving structural failure time model showed that those originally randomized to AMX0035 had a 6.9- and 10.6-month longer median survival than those originally randomized to placebo, respectively (Amylyx). Despite the small population of phase 2 trials, AMX0035 was still approved by the FDA as the third treatment for ALS on 29 September 2022, due to the severity of disease and the need for safe and effective treatment (McGinley, 2022).

A larger phase 3 trial (NCT05021536) is taking place in the United States and Europe (France, Germany, etc.) to further investigate the efficacy and safety of AMX0035 in ALS patients. A total of 600 patients will be enrolled, divided into the placebo group and AMX0035 group, and administered orally for 48 weeks. The main outcome is the change in the ALSFRS score and the number of patients with adverse events or remaining in the study drug group (Amylyx, 2021).

Methylcobalamin, the active form of Vitamin B12, is a coenzyme of methionine synthase, which is responsible for the regeneration of methionine from homocysteine. It can help eliminate neurotoxic homocysteine, which can lead to motor neuron damage and cell apoptosis by inducing excitotoxicity, oxidative stress, inflammation and mitochondrial dysfunction (Zoccolella et al., 2010). In addition, it can protect cortical neurons against glutamate neurotoxicity (Akaike et al., 1993). The effect of methylcobalamin may be dose dependent. In a rat model of sciatic nerve injury, the nerve regenerates only when used at high doses but not at low doses (Okada et al., 2010).

A phase 3, multicenter, placebo-controlled, double-blind, randomized trial conducted by Ryosuke Oki et al. evaluated the efficacy and safety of ultrahigh-dose (50 mg) methylcobalamin (Oki et al., 2022). A total of 130 Japanese patients who had been diagnosed within 1 year of ALS symptom onset were included in this study. Of these, 65 patients received methylcobalamin and 65 received placebo. After 16 weeks of treatment, the least square mean difference in the ALSFRS-R total score of the methylcobalamin group was 1.97 higher than that of the placebo group (-2.66 vs. -4.63; 95% CI, 0.44–3.50; *p* = 0.01). Clinical deterioration was reduced by 43%, and it was well tolerated during the treatment period.

SAR443820 is an inhibitor of receptor-interacting serine/threonine protein kinase 1 (RIPK1), an intracellular protein that is activated by tumor necrosis factor alpha (TNF‐α) through TNF receptor 1 and can induce neuroinflammation by increasing microglial activity and cytokine release and mediate neuronal cell death through apoptosis and necroptosis (Yuan et al., 2019; Vissers et al., 2022). The inhibition of RIPK1 was found to block oligodendrocyte death, microglial inflammation and axonal degeneration in both SOD1G93A mice and Optn−/− mice (Yuan et al., 2019). A phase 1 study showed that SAR443820 was well tolerated and could bind effectively to RIPK1((Single, 2021)). A phase 2 international clinical trial is ongoing to evaluate the safety and effectiveness of SAR443820 (Sanofi, 2022).

GDC-0134 is a dual leucine zipper kinase (DLK) inhibitor that can protect against axon degeneration and neuronal apoptosis by suppressing the DLK/c‐Jun N‐terminal kinase pathway. However, the phase 1 study showed that GDC-0134 was unsafe with ocular toxicity and not suitable for further development. A preclinical study also showed that inhibition of DLK might increase plasma neurofilament light chain (Nfl) not driven by nerve injury (Katz et al., 2022).

#### Anti-inflammatory

##### Regulation of immune cells

Masitinib is an oral selective tyrosine kinase inhibitor that can exert neuroprotection by inhibiting the activity of microglia, macrophages and mast cells in both the central and peripheral nervous systems to attenuate neuroinflammation (Mora et al., 2020). It selectively inhibits tyrosine kinase receptor colony-stimulating factor 1R (CSF-1R) to prevent microglia from proliferating. In an ALS (SOD1^G93A^) rat model, masitinib could reduce microglia proliferation and migration induced by M-CSF, decrease aberrant glial cells, inhibit neuroinflammation and prolong post-paralysis survival (Trias et al., 2016).

The results from an international, phase 2/3, placebo-controlled trial in 394 adult patients with ALS showed that masitinib combined with riluzole could significantly slow the deterioration of ALSFRS-R scores by 27% (Mora et al., 2020). The long-term overall survival analysis indicated that oral masitinib, if delivered early, could prolong survival by 2 years and decrease the mortality rate by 44% (Mora et al., 2021). Moreover, a phase-3, multicenter, placebo-controlled, double-blind, randomized trial (NCT03127267) will compare the efficacy and safety of masitinib in combination with riluzole in 495 patients with ALS from 13 countries (United States, Denmark, France, Germany, Israel, Russian, Ukraine, etc., mainly American and European cohorts), sponsored by AB Science. The main endpoint is the change in ALSFRS-R scores during the 48-week treatment (AB Science, 2017).

NP001 is a regulator of macrophages and monocytes developed by Neuraltus Pharmaceutic. It can transform macrophages back to a noninflammatory state, downregulate NF-kB expression and decrease the production of proinflammatory cytokines (Miller et al., 2022a). Although the data from a phase 2a clinical trial indicated that it may slow the disease progression of patients with higher levels of C-reactive protein (CRP) and have a good safety profile (Miller et al., 2015), the following 2b clinical trial failed to show efficacy. There were no significant differences in the change in ALSFRS-R scores and vital capacity (VC) between the placebo and NP001 groups after 6 months of treatment in 138 American and Canadian parents with elevated plasma CRP (Miller et al., 2022a). However, the post hoc analysis of the 2b trial showed that NP001 significantly slowed and even halted the progression in some cases. For patients between ages 40–65, onset within 3 years prior, and with CRP greater than 1.13 mg/L, the degression of the ALSFRS-R score was slowed down by 36%. Furthermore, the loss of VC was also slowed down by 51%, which made NP001 the first medicine to have an effect on the preservation of VC (Miller et al., 2022a) and a very promising drug for patients who respond.

Verdiperstat is an inhibitor of myeloperoxidase (MPO), which is a pro-oxidant enzyme in activated macrophages and microglia. By suppressing MPO, oxidative stress and inflammation levels could be reduced (Stefanova et al., 2012). In ALS patients, the activation of MPO is high and induces the accumulation of carboxyethylpyrrole (CEP), a peroxidation product of docosahexaenoic acid that is increased in nearly all astrocytes and microglia and may be a potential hallmark of oxidative damage in brains. However, CEP deposition in neurons differs between the subtypes, as CEP was observed in >90% of neurons only in individuals with SOD1 mutation (Xiong et al., 2022). This may be because MPO was activated due to the failure of antioxidative mechanisms and aggregation of misfolded proteins caused by the mutation of SOD1. The activation of MPO/HOCl may also facilitate ferroptosis, lipid peroxidation, and apoptosis in neurons (Peng et al., 2022). Therefore, Verdiperstat may work better in patients with SOD1 mutations.

A phase 2/3, randomized, placebo-controlled, multicenter, 24-week clinical trial using the HEALSY ALS Platform is taking place in the United States to evaluate the safety and efficacy of Verdiperstat in 167 patients, conducted by Biohaven and expected to end in November 2022. The main endpoint is the change in the ALSFRS-R score (Cudkowicz, 2020a).

Tegoprubart (AT-1501) is an antibody targeted at CD40 ligand (CD40 L), which is a membrane protein that can increase the peripheral immune response and induce neuroinflammation (Ots et al., 2022). The CD40 costimulatory pathway was found to be upregulated in the blood of 56% ALS patients, and the inhibition of CD40 L was found to delay paralysis and prolong survival in an ALS mouse model (Lincecum et al., 2010). A phase 2a study demonstrated that Tegoprubart is safe and well tolerated and could reduce key inflammatory biomarkers (including TNF-α, MCP1, EN-RAGE, CRP, etc.) dose-dependently, supporting advancing the drug into larger trials (Anelixis Therapeutics, 2020; Eledon Pharmaceuticals, 2022).

BLZ945 is also a CSF-1R inhibitor that reduces the proliferation of microglial cells and invasion of macrophages into peripheral nerves in SOD1 G93A mice (Martínez-Muriana et al., 2016). An open-label phase 2 trial is ongoing to evaluate the safety and brain microglial response of BLZ945 (Novartis Pharmaceuticals, 2019).

##### Enhanced regulatory T cells (tregs)

Tregs normally suppress proinflammatory responses, and the lack of functionally suppressive Tregs increases inflammation. Previous studies have shown that decreased levels of Tregs were correlated with faster disease progression, increased disease severity and lower survival in patients with ALS (Henkel et al., 2013). The expression of FOXP3 and the number of Tregs decrease greatly in rapid-progressing patients, and the proliferation and suppressive function of Tregs is regained when treated with interleukin (IL)-2 and rapamycin *ex vivo* (Alsuliman et al., 2016; Thonhoff et al., 2018). Therefore, enhancing the numbers and function of Tregs may have therapeutic benefits for patients.

Proleukin (Aldesleukin) is a recombinant IL-2 that can induce the proliferation of Tregs and enhance their function. IL-2 is a key cytokine for the generation, activation and survival of Tregs. Low-dose IL-2 was found to expand Tregs selectively and safely in mice and humans (Ye et al., 2018). A phase 2a study validated that aldesleukin was well tolerated and could expand the numbers and frequency of Tregs. The expression of CD25 was also found to increase on Tregs, which may enhance the sensitivity of Tregs to administered and endogenous IL-2 and increase the efficacy (Camu et al., 2020). Additionally, it has become an orphan drug in America and Europe. Another phase 2 clinical trial (MIROCALS) is taking place in the United Kingdom and France to further evaluate the efficacy and safety of aldesleukin (Centre Hospitalier Universitaire de Nīmes, 2017). The trial has been completed, but the outcomes have not yet been shared.

Dimethyl fumarate is an oral immunomodulatory agent that can increase the number of Tregs and suppress proinflammatory T cells by binding to the transcription factor nuclear factor erythroid-derived 2-like 2 (Nrf2). However, according to data from a phase 2 trial, it failed to slow the decline of ALSFRS-R or extend survival. Only the neurophysiological index (NI), a potent biomarker of disease progression in ALS, was decreased, although not significantly. The decline in the ALSFRS-R in both groups was relatively slow, so the chance of its benefit on fast-progressing patients was not excluded (Vucic et al., 2021a).

##### Complement inhibition

Although the exact cause of ALS is still unclear, improper activation of the complement system may play critical roles in the development and progression of ALS (Dalakas et al., 2020). The complement system is dysregulated in patients with ALS. C1q, C3 and C5a were upregulated and deposited on neuromuscular junctions (NMJs), glial cells or neurons in both mice and patients with ALS ((Carpanini et al., 2019)). Thus, the complement system could be a potential target for ALS.

Pegcetacoplan is a complement component 3 (C3) inhibitor that can prevent the cleavage of C3 and attenuate the recruitment of inflammatory cells. C3 inhibitors may also reduce the deposition of C3b in axons and synaptic bodies in nerve cells (Hoy, 2021; Lamers et al., 2022). However, in SOD1^G93A^ ALS mice, deletion of the C3 gene did not affect progression, so the benefit of c3 inhibitors is still unclear (Lobsiger et al., 2013). A phase 2 study is taking place to assess the efficacy and safety of Pegcetacoplan in ALS (Apellis Pharmaceuticals, 2020).

ANX005 is a recombinant antibody against complement component 1q (C1q). In SOD1^G93A^ mice, it decreased C1q in plasma, spinal cord and muscle tissue and preserved synaptic connectivity of neuromuscular junctions, reducing neuronal damage (Andrews-Zwilling et al., 2022). However, the deletion of the C1 gene did not affect disease onset or progression. C1q affects the maintenance, not the loss, of synaptic connections in SOD1^G93A^ ALS mice (Lobsiger et al., 2013). A phase IIa study is ongoing to evaluate the safety and tolerability of ANX005 (Annexon, 2021).

Zilucoplan could bind and inhibit complement component 5 (C5). The inhibition of C5 was found to prolong the survival of SOD1^G93A^ ALS mice (Woodruff et al., 2014). However, due to the lack of benefit, the phase 2/3 clinical trial was stopped early (Figueiredo).

Ultomiris (ravulizumab) is also a C5 inhibitor. Similar to zilucoplan, a phase 3 trial enrolled 382 patients and stopped early due to the lack of efficacy (AstraZeneca, 2021). Therefore, C5 may not be a potential therapeutic target for ALS.

##### Inhibition of proinflammatory cytokines

MN-166 (ibudilast) is a small molecule that can inhibit phosphodiesterase type-4 (PDE4), proinflammatory cytokines, and macrophage migration inhibitory factor (MIF). It can also promote the production of anti-inflammatory cytokines and neurotrophic factors (Oskarsson et al., 2021a; Babu et al., 2021). It was recently found that MN-166 can also enhance the autophagy of SOD1 and TDP-43 aggregation mediated by the transcription factor EB (Chen et al., 2020a). The safety and tolerability were validated in a phase 2 trial. After 6 months of treatment, the rate of responders on the ALSAQ-5 score of the MN-166 group was greater than that of the placebo group (29.4% vs. 17.6%) (MediciNova, 2014; Bautz, 2019). A phase 2b/3, multicenter, two-arm, randomized, double-blind clinical trial is taking place in the United States and Canada to evaluate the efficacy, safety and tolerability of MN-166. A total of 230 patients will be included, randomized 1:1 between placebo and MN-166 for 12 months of treatment, followed by a 6-month open-label extension phase. The main outcome is the change in ALSFRS-R scores (Oskarsson et al., 2021a).

Tocilizumab is an antibody against IL-6 receptor (IL-6R). It can block both classical IL-6 signaling and IL-6 trans-signaling and IL-6 trans-signaling mediated by IL-6R (Milligan et al., 2021). IL-6 levels were elevated in the serum of patients with ALS and only increased in the cerebrospinal fluid (CSF) of those who also had the IL-6R^358^Ala variant. In addition, carriers exhibited a faster progression rate than noncarriers (Wosiski-Kuhn et al., 2019), and only in carriers was serum IL-6 negatively correlated with patient prognosis (Wosiski-Kuhn et al., 2021). Hence, tocilizumab may have a better benefit for patients with the IL-6R^358^Ala variant, which is found in European (40%) and Native American (50%) populations (Wosiski-Kuhn et al., 2019). Data from a phase 2 clinical trial showed that tocilizumab was well tolerated and could reduce C-reactive protein concentrations in patients with ALS. In addition, CRP was lower in patients with more *IL-6R C* alleles, suggesting that carriers may benefit most from the drug (Milligan et al., 2021).

#### Anti-excitotoxicity agent

Memantine is a noncompetitive antagonist of the N-methyl-D-aspartate receptor (NMDAR), an important ionotropic glutamate receptor. The accumulation of glutamate in the extracellular space in ALS leads to the increased activation of NMDARs. This leads to the sustained influx of calcium into neurons, which further causes oxidative stress to the mitochondria and neuronal death (Armada-Moreira et al., 2020). Thus, inhibition of NMDARs may decrease the excitotoxicity of glutamate. Memantine was found to delay the progression and prolong the survival of ALS SOD1^G931^ mouse models (Wang and Zhang, 2005) and decreased levels of CSF tau in patients (Levine et al., 2010). Data from a phase 2/3 study, which enrolled 63 patients in Portugal, indicated that during 12 months of treatment with memantine 10 mg twice daily (BID), the drug was well tolerated but with no significant difference in ALSFRS score (de Carvalho et al., 2010). Another phase 2 clinical trial took place in the United States to evaluate the safety and efficacy of memantine 20 mg BID. The trial finished in 2021, but the outcome has not yet been shared (University of Kansas Medical Center, 2014). In England, a phase 2/3 systematic multiarm adaptive randomized trial is ongoing, with 177 patients taking 10 mg/5 ml memantine hydrochloride, 177 patients taking 100 mg/5 ml trazodone hydrochloride and 177 patients taking placebo. The main endpoint is the change in the ALSFRS score during 18 months of treatment (Wong et al., 2022).

Mexiletine is a cardiac antiarrhythmic agent that has also been found to suppress neuronal hyperexcitability by blocking sodium channels and slowing conduction of nerve impulse (Weiss et al., 2021). A phase 2 trial demonstrated that it could reduce hyperexcitability (Weiss et al., 2021). A systematic review indicated that mexiletine could not prolong survival, but it could significantly reduce the severity and frequency of muscle cramps (Adiao et al., 2020). Therefore, although mexiletine may not prolong survival, it could be used to treat muscle cramps in patients with ALS.

Ezogabine (retigabine) is an antiepileptic medicine that can reduce neuronal excitability by activating KCNQ channels. It is known that steady-state potassium currents are decreased in ALS motor neurons and that the activation of Kv7.2/7.3, a potassium channel encoded by KCNQ2 and KCNQ3 and a main component of the neuron M-current, can stabilize membrane potential and block neuron hyperexcitability. A high-throughput GCaMP screen using human ALS cells also demonstrated that the Kv7 ion channel is a strongly overrepresented drug target for excitotoxicity (Huang et al., 2021). Ezogabine reduced excitability and prolonged survival of SOD1^A4V/+^ ALS neuron cells *in vitro* (Wainger et al., 2014). In a phase 2 trial with 65 patients, it was found to be well tolerated and could also increase short-interval intracortical inhibition as well as lower cortical and spinal motor neuron excitability. However, due to lack of selectivity, adverse events were common (Wainger et al., 2021). It can also interact with the GABAa receptor (van Rijn and Willems-van Bree, 2003). Therefore, a more selectively Kv channel-targeted drug is needed. QurAlis has developed a new drug, QRL-101, which is more selective than retigabine and does not bind the GABA receptor and will begin a trial to evaluate it (QurAlis, 2022).

Penicillin G/hydrocortisone may suppress gamma aminobutyric acid (GABA). In three Caucasian patients, it was reported that their symptoms were improved with the treatment (Tuk et al., 2017). However, data from a 16-person phase 2 trial showed that it could not halt the progression of disease (Van Es et al., 2020).

Perampanel is an inhibitor of α-amino-3-hydroxy-5-methyl-4-isoxazolepropionic acid (AMPA) receptors that may induce chronic excitotoxicity of motor neurons and was identified as a main target for excitability using a high-throughput GCaMP screen in human ALS patients (Oskarsson et al., 2021b; Huang et al., 2021). It is mainly used for epilepsy. However, a 66-patient, phase 2 clinical trial showed that perampanel was not well tolerated and was significantly associated with a decline in the ALSFRS score (Aizawa et al., 2022). Non-AMPA antagonism against voltage-gated sodium channels and M-type potassium currents may exert negative effects (Lai et al., 2019).

#### Integrated stress response regulation

The ISR is a cellular central signaling network that senses stress situations through four kinases and remodels translation and transcription to restore homeostasis in the cell. However, under chronic or excessive stress, ISR can also promote cell death. ISR is activated in ALS and can further promote the initiation of neurotoxicity after induction by stress (Marlin et al., 2022). Thus, ISR may be a potential target for the treatment of ALS.

Trazodone is an approved drug for depression that is also found to inhibit protein kinase RNA-like endoplasmic reticulum kinase (PERK) and reduce the formation of stress granules (Wong et al., 2022). Inhibition of PERK was found to reduce TDP-43 toxicity in ALS flies and rat models (Kim et al., 2014). In a cellular model with repeated C9orf72 mutations, trazodone inhibited the production of toxic proteins (Westergard et al., 2019). A phase 2/3 systematic multiarm adaptive randomized trial is taking place in England to evaluate the efficacy of both trazodone and memantine (Wong et al., 2022).

Guanabenz is a PPP1R15A/PP1c phosphatase complex inhibitor. It could disrupt the complex between PPP1R15A and PP1 to slow the dephosphorylation of eIF2α-P, prolong ISR and exert protection against misfolding (Das et al., 2015). In a phase 2 trial, Guanabenz was found to significantly slow ALS progression. However, due to the α2-adrenergic activity of Guanabenz and the rate of adverse events (low blood pressure), the drop-out rate was high, which discourages further development for Guanabenz (Dalla Bella et al., 2021).

IFB-088 Similar to guanabenz, IFB-088 can also prolong ISR to protect against cellular stress in stressed cells by selectively suppressing the PPP1R15A/PP1c phosphatase complex while leaving normal cells unchanged (Das et al., 2015). It has been granted orphan drug designation by the FDA. Data from a phase 1 study with 72 healthy males showed that it was well tolerated. It was safer than Guanabenz, as IFB-088 did not have α2-adrenergic activity (BioScience, 2018). A phase 2 study is ongoing to determine the safety and efficacy of IFB-088 in ALS patients (InFlectis BioScience, 2022).

ABBV-CLS-7262 can inhibit ISR by activating eukaryotic initiation factor 2B (eIF2B), a key regulator of ISR. It may restore protein production and clear TDP-43-containing stress granules. A phase 1 study is ongoing to investigate the safety and pharmacokinetics of ABBV-CLS-7262 (Calico Life Sciences LLC, 2021).

DNL343 is also an eIF2B activator. A phase 1b study is taking place to evaluate the safety and pharmacokinetics of DNL343 (Denali Therapeutics, 2021).

Overall, these five drugs regulate the ISR through three mechanisms: they regulate ISR kinases directly (Trazodone), they regulate dephosphorylation of phospho-eIF2a (Guanabenz and IFB-088), and they regulate eIF2b, which is downstream of the pathway (ABBV-CLS-7262 and DNL343). Both prolonging ISR and inhibiting ISR show potent benefits for ALS, and they may work in certain subsets of ALS and aggravate the disease in other subsets. In addition, the ISR mechanism differs between the ALS subsets (SOD1, C9orf72 and TDP43, etc.) and animal models. The heterogeneity in the ISR response in ALS patients may affect the success of clinical trials (Marlin et al., 2022).

#### Neurotrophic factors and neuroprotection

CNM-Au8 is a gold nanocrystal suspension that has been demonstrated to be an efficient catalyst for energy metabolism. By promoting the oxidation of NADH to NAD+ and the production of ATP, it can restore energy in brain cells and alleviate energetic dysregulation, which plays a key role in the progression of ALS (Vucic et al., 2021b). It can also reduce the accumulation of TDP-43 aggregates in the cytoplasm (Ho et al., 2022). Directly addressing energetic dysregulation in ALS patients is a paradigm shift. A phase 2 study enrolled 45 Australian patients and evaluated the safety and efficacy of CNM-Au8. Although it failed to reach the primary endpoint in the change in the motor unit number index (MUNIX) in the original double-blind portion, in the limb onset subgroup, there was a significant treatment effect in the MUNIX group at week 12 (*p* = 0.057). At week 36, CNM-Au8 also slowed the progression of disease (*p* = 0.125), reduced the patients with a 6-point decline in the ALSFRS-R score (*p* = 0.035), and improved the quality of life measured by ALS Specific Quality of Life (*p* = 0.018). The unadjusted Kaplan‒Meier analyses for the following open-label showed that CNM-Au8 reduced the risk of death by 70% compared with those initially treated with placebo (log-rank HR, 0.301; 95% CI, 0.122–0.742; *p* = 0.0143) (Meglio, 2022a). A larger, placebo-controlled, phase 2/3 study is taking place in the United States using the HEALEY ALS Platform to further investigate the safety and efficacy of CNM-Au8. A total of 161 patients were randomly allocated to CNM-Au8 or placebo in a 3:1 ratio. The main outcome is the change in the ALSFRS-R score after 24 weeks of treatment (Cudkowicz, 2020b).

Pridopidine is a sigma-1 receptor (S1R) agonist that may enhance the secretion of brain-derived neurotrophic factor and glial cell line-derived neurotrophic factor, exhibiting potential neuroprotective effects. It can restore NMJ activity, modulate AT deficits and reduce neuron loss in SOD1^G93A^ neuromuscular cocultures by activating the ERK pathway mediated by S1R (Ionescu et al., 2019). In a SOD1^G931^ mouse model, pridopidine reduced SOD1 aggregation and ameliorated muscle fiber atrophy (Ionescu et al., 2019). Autophagy is enhanced as the activation of SIGMAR1/S1R by pridopidine can chaperone the NP protein POM121, which recruits KPNB1 to transport TFEB into the nucleus. Therefore, pridopidine may ameliorate the TFEB transport deficit in the C9orf72 subtype of amyotrophic lateral sclerosis-frontotemporal lobar degeneration (ALS-FTD), where the mutation damages the nucleocytoplasmic transport of TFEB (Wang et al., 2022). It has been approved as an orphan drug for ALS in the US and Europe. A phase 2/3, placebo-controlled study is ongoing in the United States to evaluate the safety and efficacy in 163 ALS patients. The main endpoint is the ALSFRS-R score after 24 weeks of treatment (Cudkowicz, 2020c).

ILB^®^ is a novel modified low molecular weight dextran sulphate that can elevate the level of hepatocyte growth factor, a neurotrophic and myogenic factor that can protect motor neurons and muscle cells and reduce inflammation and oxidative stress. It may also improve tissue energy metabolism. It was granted Orphan Drug Designation by the European Commission. Data from a phase 2 open label clinical trial enrolled 13 patients, and ILB^®^ was reported to be well tolerated by patients. After 5 weeks of treatment, the ALSFRS-R score improved from 70.61 ± 13.91 to 77.85 ± 14.24, and the alleviation of the progression lasted 3–4 weeks, which suggested that ILB^®^ may have the potential to halt and even reverse the symptoms of ALS (Logan et al., 2022).

Granulocyte-colony stimulating factor (G-CSF) is a growth factor that exerts neuroprotective activities by inhibiting apoptosis, inducing neurogenesis, mobilizing hematopoietic stem cells (HSCs) and reducing inflammatory activity. G-CSF prolonged the survival of SOD1^G93A^ mouse models and was safe and well tolerated in clinical trials (Vafaei Mastanabad et al., 2022). A phase 2 study is ongoing to further investigate the efficacy and safety of G-CSF (Salamone et al., 2020).

Fasudil is an inhibitor of the Rho kinase (ROCK) protein approved for subarachnoid hemorrhage. It may counteract neuronal apoptosis, foster neuronal regeneration and regulate microglia in animal models of neurodegenerative disorders (Koch et al., 2018). Three patients were treated with fasudil under compassionate use. During the 4-week treatment, the ALSFRS-R scores remained stable, and no adverse side effects were reported (Koch et al., 2020). A phase 2a clinical trial was conducted in Europe to confirm the benefits of fasudil in 120 patients (University Medical Center Goettingen, 2019).

Inosine is an endogenous antioxidant that may protect against oxidative stress. Urate, the metabolite of inosine, is an endogenous antioxidant and potential neuroprotectant. Its level is lower in patients with ALS, and a high urate level is associated with improved survival (Basile et al., 2022). Furthermore, increased inosine metabolism may be protective in ALS. In C9orf72 and some sporadic patients with ALS, adenosine deaminase is decreased in astrocytes, which leads to adenosine metabolism dysfunction, reduces bioenergetic output and causes toxicity in ALS astrocytes. Inosine supplementation *in vitro* was found to increase glycolytic flux, bioenergetic capacity and survival of motor neurons by reducing induced astrocyte-mediated toxicity (Allen et al., 2019). In a phase 2 study, inosine was well tolerated by patients. Serious adverse events were reported in 28.57% (4/14) of patients receiving inosine and 11.11% (1/9) of those receiving placebo, including device-related infection, acute kidney injury, nephrolithiasis and laryngospasm (Massachusetts General Hospital, 2017).

#### Anti-aggregation

##### Protein aggregation antibody

AP-101 is a human antibody against misfolded and aggregated SOD1 protein. It has been found to attenuate motor symptoms and increase overall survival in mouse models. A phase 1 study demonstrated that AP-101 was well tolerated by patients with ALS (Genge et al., 2021). A phase 2a study is ongoing to evaluate the safety, tolerability, pharmacokinetics, and pharmacodynamics of AP-101 in 63 patients (AL-S Pharma, 2021).

##### Enhanced autophagy

Colchicine can induce the expression of heat shock protein B8, which can enhance autophagy to eliminate misfolded SOD1 and TDP-43 aggregation as well as C9ORF72-related aggregated poly-dipeptides (Mandrioli et al., 2019). A phase 2 clinical trial is ongoing to evaluate the efficacy of two different doses of colchicine in patients with ALS (Mandrioli et al., 2019).

Arimoclomol can also increase the production of heat shock protein to enhance autophagy. However, a phase 3 study failed to reach its primary and secondary goals. Arimoclomol did not slow the progression or extend the survival of 245 patients after 18 months of treatment (KemPharm Denmark A/S, 2018; Inacio, 2021).

Rapamycin is a mTOR inhibitor. By inhibiting mTORC1, a protein complex that can inhibit autophagy, rapamycin can promote the production of autophagosomes from the phagophore to enhance autophagy. It can also increase Treg cells to protect against inflammation (Mandrioli et al., 2018). A phase 2 study showed that 18 weeks of rapamycin treatment increased the level of Tregs, decreased inflammasomes and improved quality of life. The change in the ALSFRS-R score per month in the rapamycin group was 0.28 points lower than that in the placebo group (-1.20 vs. -1.48) (Azienda Ospedaliero-Universitaria di Modena, 2017; ENCALS, 2022).

Monepantel is also a mTOR inhibitor. A phase 1 clinical trial is ongoing to evaluate the safety and pharmacokinetics of monepantel (PharmAust Ltd, 2022).

Tamoxifen was found to enhance autophagy through both mTOR-dependent and mTOR-independent pathways to attenuate pathologic TDP-43 in mouse models (Wang et al., 2013). However, in a 1/2 clinical trial in Taiwan, the decline in the ALSFRS-R score slowed for only 6 months. There was no significant difference in the ALSFRS-R score between the tamoxifen group and the placebo group (Chen et al., 2020b).

Bosutinib is a selective inhibitor of Src/c-Abl kinase approved for chronic myelogenous leukemia. It can promote autophagy and reduce the amount of misfolded mutant SOD1 protein. It also prolonged the survival of ALS iPSC-derived motor neurons *in vitro* (Imamura et al., 2019). An open-label phase 1 trial is ongoing to investigate the safety and tolerability of bosutinib at four different doses. Among nine Japanese patients, five stopped progressing, while the other four continued to progress after 12 weeks of treatment (Imamura et al., 2022). A phase 1/2 clinical trial is ongoing to further evaluate the efficacy and safety of bosutinib in 25 ALS patients for 24 weeks (Haruhisa, 2022).

SLS-005 can cross the blood‒brain barrier and boost autophagy by activating the transcription factor EB and the FOXO1 transcription factor, key factors in the expression of autophagy genes (Castillo et al., 2013; Pupyshev et al., 2022). It can reduce abnormal cellular protein aggregation and prolong survival in SOD1 transgenic mouse models (Castillo et al., 2013) and has received orphan drug designation in the United States and Europe. A phase 2/3 study is ongoing in the United States using the HEALEY ALS Platform to further evaluate the safety and efficacy of SLS-005. A total of 160 patients were allocated randomly to active treatment with SLS-005 or treatment with placebo at a 3:1 ratio. The main endpoint is the change in the ALSFRS-R score at week 24. Secondary endpoints include SVC, hand-held dynamometry and survival (Cudkowicz, 2021).

Withaferin A is a natural compound extracted from Withania somnifera. It can reduce inflammation and enhance autophagy by inhibiting NF-κB activity. In a TDP-43 G348C mouse model, it alleviated TDP-43 pathology and increased the levels of the autophagic marker LC3BII (Kumar et al., 2021). A double-blind, placebo-controlled, phase 2 clinical trial is ongoing to determine the safety of Withania somnifera in patients with ALS (Sunnybrook Health Sciences Centre, 2021).

3K3A-APC is a modified activated protein C that may repair the blood‒brain barrier and inhibit the activation of microglia while lacking its anticoagulant properties (Zhong et al., 2009). It can also restore the formation of autophagosomes in both C9ORF72 ALS/FTD and sporadic ALS iMNs by activating protease-activated receptor 1 (PAR1) (Shi et al., 2019). In a mouse model, 3K3A-APC reduced the synthesis of SOD1 and delayed its progression (Zhong et al., 2009). An open-label phase 2 trial is ongoing to investigate the safety and efficacy of 3K3A-APC (Macquarie University, 2021).

AL-001 (latozinemab) AL-001 is a monoclonal sortilin antibody designed to increase progranulin levels, a protein that can stimulate the removal of TDP-43 through autophagy (Chang et al., 2017). It was well tolerated and elevated progranulin above physiological levels in patients with C9orf72-mutant frontotemporal dementia (Alector, 2022). A placebo-controlled, phase 2 study is ongoing to evaluate the safety, tolerability, pharmacokinetics and pharmacodynamics of AL-001 in patients with C9orf72-associated ALS (Alector, 2021).

##### Other mechanisms

Tideglusib is a non-ATP competitive inhibitor of glycogen synthase kinase (GSK)-3β, a protein kinase involved in TDP-43 phosphorylation. It reduced abnormal phosphorylation of TDP-43 and efficiently decreased the accumulation of cytosolic TDP-43 in lymphoblasts from patients with sporadic ALS. It also inhibited the phosphorylation of TDP-43 in TDP-43 transgenic mice (Martínez-González et al., 2021). A phase 2 study is ongoing to evaluate the safety and tolerability of tideglusib (Geneva University Hospitals, 2021).

#### Gene therapy

Tofersen (BIIB067) is a SOD1 antisense oligonucleotide. It reduced the synthesis of SOD1 protein by degrading SOD1 mRNA. In a phase 1/2 ascending-dose trial, tofersen reduced the levels of SOD1 proteins from baseline by 36% in the CSF of patients treated with 100 mg tofersen and 3% in placebo groups. Most adverse events were mild to moderate, including headache, procedural pain, and postlumbar syndrome related to lumbar puncture (Miller et al., 2020).

A phase 3, randomized, double-blind, placebo-controlled trial (VALOR) evaluated the efficacy and safety of tofersen in 108 patients in America, Europe, and Asia. Seventy-two patients received tofersen 100 mg and 36 patients received placebo for 24 weeks, followed by an open-label extension for up to 236 weeks. The main endpoint was the change from baseline to week 28 in the ALSFRS-R, which did not meet the statistical significance. For the secondary endpoints, although in the faster-progression subgroup, CSF SOD1 protein (HR [95% CI] 0.62 [0.49–0.78]) and plasma Nfl (HR [95% CI] 0.33 [0.25 to 0.45]) were reduced, there was no significant difference in SVC or handheld dynamometry. However, the open-label extension showed that earlier tofersen initiation led to better measurement outcomes. At week 52, the change in the ALSFR-R score of early-start participants was 3.5 points lower than that of delayed-start participants (95% CI, 0.4–6.7). The decline in SVC and handheld dynamometry were also slowed in earlier initiation of tofersen. This suggested that a trial duration of more than 28 weeks may be required to further evaluate whether tofersen is effective (Miller et al., 2022b).

Another phase 3 study (ATLAS) is ongoing to evaluate the effect of tofersen when initiated early in 150 presymptomatic carriers of SOD1 mutation with elevated neurofilaments in America, Europe and Asia. The main endpoint is the proportion of participants who showed clinical manifestations of ALS within 12 months (Benatar et al., 2022).

ION363/Jacifusen is an antisense oligonucleotide targeted at FUS mRNA (Fused in Sarcoma Mutations). Mutation of FUS, an RNA processing protein, can lead to motor neuron degeneration through toxic gain of function (Sharma et al., 2016). By silencing the expression of FUS, ION363 reduced the levels of FUS and prevented the loss of motor neurons (Korobeynikov et al., 2022). In one patient with ALS, it also reduced the FUS protein considerably in the brainstem tissue (Korobeynikov et al., 2022). A phase 3 trial is ongoing to determine the safety and efficacy of ION363 in 77 patients in American and European cohorts with FUS mutations. Patients will be administered ION363 or placebo double-blindly every 4–12 weeks for 61 weeks, followed by an 85-week open-label treatment. The main endpoints are the change in the ALSFRS-R score and ventilation assistance-free survival (Ionis, 2021).

WVE-004 is an antisense oligonucleotide with a C9ORF72 mutation that mediates the degradation of C9ORF72 mRNAs containing the G4C2 repeat, a hexanucleotide expansion that may result in an increase in abnormally translated dipeptide repeat proteins (DPRs) and cause neurotoxicity (Lee et al., 2017). An ongoing phase 1/2 clinical trial showed that after 3 months of treatment, WVE-004 reduced poly-GP DPRs in cerebrospinal fluid, a key biomarker for C9ORF72-associated ALS patients (Wave Life Sciences Ltd, 2021; Meglio, 2022b).

BIIB078 is an antisense of C9orf72 gene mRNA. As there was no difference in ALSFRS-R score, SVC, hand-held dynamometry, or the Iowa oral pressure Instrument between the BIIB078 group and placebo group in the phase 1 trial. The development of the drug had been discontinued (Biogen, 2020; Hoffman, 2022).

BIIB105 is an ataxin-2 antisense that can degrade ataxin-2 mRNA and reduce the level of ataxin-2 protein. The decrease in ataxin-2 reduced aggregation of TDP-43 and increased the survival of TDP-43 transgenic mice (Biogen, 2020). A phase 1 clinical trial is ongoing to evaluate the safety and tolerability of BIIB105 (Biogen, 2020).

APB-102 is a recombinant AAVrh10 vector that can produce an anti-SOD1 microRNA. This can bind to SOD1 mRNA and reduce the production of SOD1 protein. It has received orphan drug status from the FDA. A phase 1/2 trial is ongoing to evaluate the safety, tolerability, and efficacy of APB-12 (Pinto, 2021).

Engensis (VM202) Engensis can deliver the hepatocyte growth factor (HGF) gene to the nerve and nerve-supporting cells through the plasmid. The increased expression of HGF, a neurotrophic factor, was found to lessen motor neuron degeneration and increase the life span of ALS mice (Sun et al., 2002). Data from a phase 2a study suggested that Engensis was well tolerated by patients (Helixmith, 2022).

#### Others

Reldesemtiv is a fast skeletal muscle troponin activator that can increase muscle strength. It selectively binds the troponin complex, slows the release of calcium from troponin C, and sensitizes the sarcomere to calcium. This makes the force-calcium relationship of muscle fibers and force-frequency relationship of a nerve-muscle pair shift leftwards, increasing the muscle’s response to neural input and enhancing strength at submaximal stimulation frequencies (Collibee et al., 2021; Shefner et al., 2021). The restoration of skeletal muscle function may preserve the integrity of NMJs and slowing the impairment of motor neurons (Lepore et al., 2019).

Reldesemtiv is more potent than the first-generation fast skeletal muscle troponin activator tirasemtiv, which shares the same mechanism (Li et al., 2021), as it does not cross the blood‒brain barrier, is associated with milder adverse events, and the doses are lower (Collibee et al., 2021). The results from a phase 2 study showed that Reldesemtiv was well tolerated. Although the primary efficacy analysis failed to reach statistical significance, the decline in SVC, ALSFRS-R and muscle strength mega-score of the Reldesemtiv group was slower than that of the placebo group. As the treatment effect was stronger in those with rapid disease progression and less disease duration, more rapid progressors will be included in the subsequent phase 3 study. In addition, the effect still lasted for 4 weeks after the active treatment stopped (Shefner et al., 2021).

A phase 3, multicenter, randomized clinical trial was conducted in Europe and America to evaluate the effect of reldesivir. A total of 555 patients with symptoms for less than 24 months are randomized to receive 600 mg reldesemtiv or placebo every day for 24 weeks, followed by an active drug period, where all the patients will receive reldesemtiv at 300 mg or 600 mg dose each day for the next 24 weeks. The main endpoint is the change in the ALSFRS-R score at week 24 (Cytokinetics, 2021).

## Discussion

**FIGURE 1:**
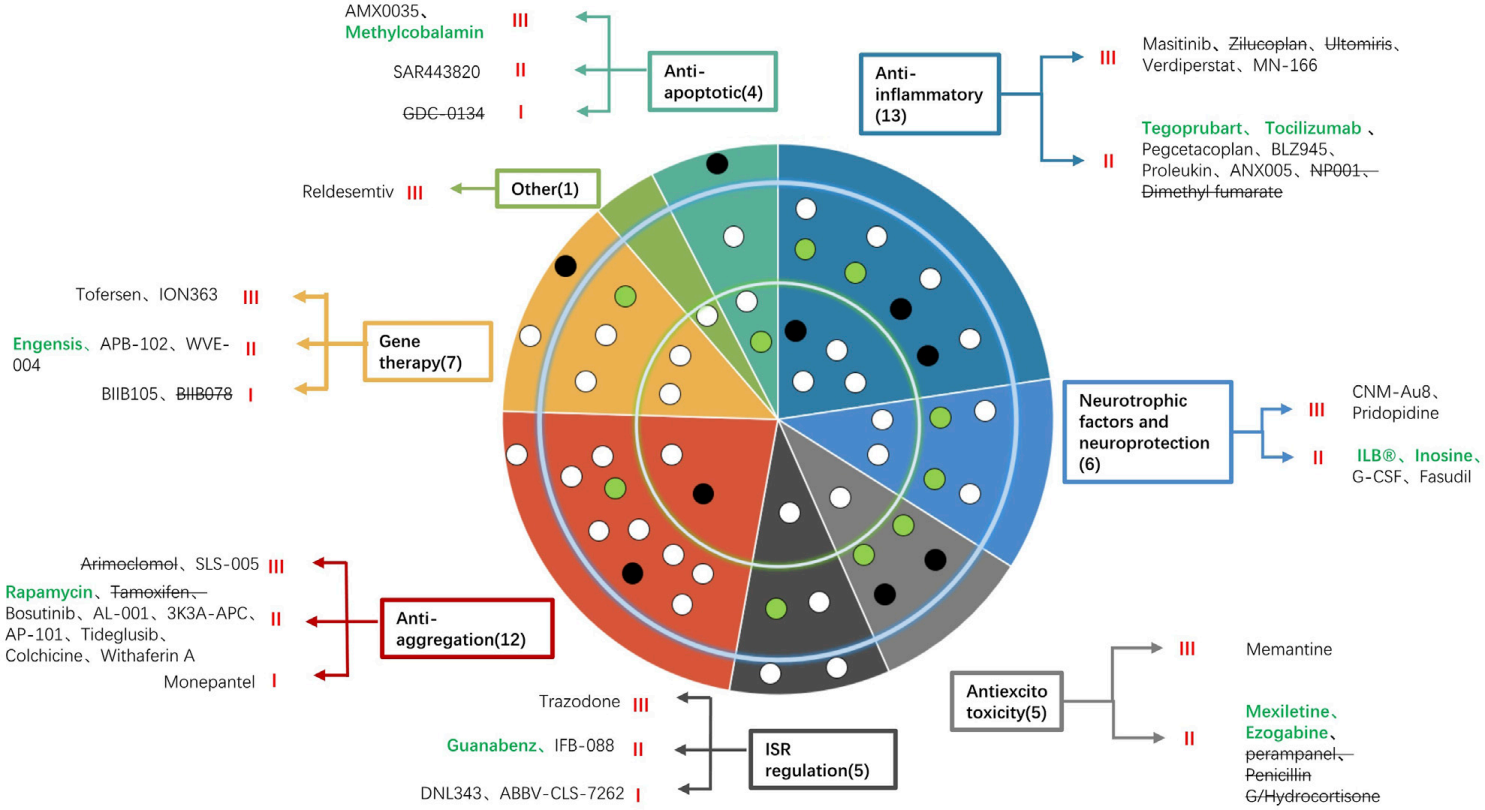
Summary of the 53 new drugs under investigation for the treatment of ALS classified by their mechanism of action and included in a concentric circle. The outer ring indicates six drugs in phase 1, the middle ring indicates 31 drugs in phase 2, and the inner ring indicates 16 drugs in phase 3. Each color indicates a mechanism (anti-apoptotic, anti-inflammatory, anti-excitotoxicity, regulation of ISR, neurotrophic factors and neuroprotection, anti-aggregation, gene therapy and other mechanisms). The names of drugs according to different mechanisms are listed in the blank. Green words indicate drugs whose trial succeeded, black words indicate that the trial was ongoing, and words with strikethroughs indicate that the trial failed. Each dot represents a drug. White dots are drugs whose trials are ongoing, black dots are drugs whose trials failed, and green dots are drugs whose trials have positive results.

In this review, we have summarized 53 new drugs that have been assessed in clinical trials from 2020 to 2022 (Figure 1). For anti-apoptotic drugs, high-dose methylcobalamin was found to reduce deterioration by 43% in a phase 3 study in Japan (Oki et al., 2022). AMX0035 was recently approved by the FDA because it slowed the decline in the ALSFRS-R score and the risk of death by 44%, prolonged tracheostomy/PAV-free survival (Paganoni et al., 2022) and prolonged survival by 10.6 months (Amylyx).

Most of the new drugs exert their protective effects by inhibiting inflammation. Among these anti-inflammatory drugs, 3 phase 3 trials are ongoing to evaluate the efficacy of masitinib (AB Science, 2017), MN-166 (Oskarsson et al., 2021a) and verdiperstat (Cudkowicz, 2020a). Although NP001 failed the phase 2b study, it still efficiently slowed the decline in the ALSFRS-R score and SVC of patients between 40 and 65 (Miller et al., 2022a). For anti-excitotoxicity drugs, memantine is being tested in England in a phase 3 study (Wong et al., 2022). Another phase 3 study also tested the efficacy of trazodone (Wong et al., 2022). For neuroprotective drugs, CNM-Au8 created a new pathway for treatment by catalyzing energy metabolism efficiently. It slowed the progression, improved the quality of life and reduced the risk of death by 70% in a phase 2 study (Meglio, 2022a). A phase 3 study is ongoing to further test the efficacy (Cudkowicz, 2020b). Pridopidine was also evaluated in a phase 3 study (Cudkowicz, 2020c). For drugs that reduce protein aggregation, only SLS-005, an activator of transcription factor EB and FOXO1 transcription factor, was tested in a phase 3 study (Cudkowicz, 2021).

Gene therapy offers new hope for the treatment of ALS. Two phase 3 trials are ongoing to evaluate the efficacy of IONN363, an antisense oligonucleotide of FUS mRNA (154), and tofersen, an antisense oligonucleotide against SOD1 (151). Tofersen improved SVC and hand-held dynamometers and greatly reduced CSF SOD1 protein and plasma Nfl. It was also found to have a better effect when used earlier (Biogen, 2021). For other mechanisms, reldesemtiv is being tested in a phase 3 trial.

Overall, among 53 new drugs, six were trialed in a phase 1 study, 31 were trialed in a phase 2 study, and three failed in a phase 3 study and stopped further development. The remaining 13 drugs are being trialed in phase 3 studies, including methylcobalamin, masitinib, MN-166, verdiperstat, memantine, AMX0035, trazodone, CNM-Au8, pridopidine, SLS-005, IONN363, tofersen and reldesemtiv. Among them, five drugs had promising evidence of efficacy in phase 2 or phase 2/3 trials, including high-dose methylcobalamin, a coenzyme of methionine synthase that helps eliminate neurotoxic homocysteine developed by Eisai; masitinib, an oral selective tyrosine kinase inhibitor that inhibits inflammatory cells developed by AB Science; AMX0035, a dual UPR-Bax apoptosis inhibitor developed by Amylyx; CNM-Au8, an efficient catalyze for energy metabolism developed by Clene Nanomedicine; and tofersen, a SOD1 antisense oligonucleotide developed by Biogen.

High-dose methylcobalamin, masitinib and AMX0035 were found to slow the decline in the ALSFRS-R score by 43% (Oki et al., 2022), 25% (Mora et al., 2020), and 25% (Paganoni et al., 2020), respectively. Masitinib, AMX0035, and CNM-Au8 decreased the risk of death by 44% (AB Science, 2017), 44% (Paganoni et al., 2022), and 70% (Meglio, 2022a), respectively. CNM-Au8 also slowed the progression (*p* = 0.0124), decreased the percentage of patients with ALSFRS-R 6-point decline (*p* = 0.035) and improved quality of life (*p* = 0.018) (Meglio, 2022a). Although tofersen did not significantly improve ALSFRS-R, it reduced SOD1 protein by 38% and Nfl by 67% and improved SVC and HHD (Biogen, 2021). All of these drugs were well tolerated by patients. Thus, due to the potent therapeutic effect, larger phase 3 trials are ongoing to further evaluate the efficacy of these drugs. Furthermore, of these five drugs, AMX0035 is the most promising and has been the third medicine officially approved by the FDA for the treatment of ALS (McGinley, 2022).

The characteristics of the study population may affect the results of the clinical trial. The complex and heterogeneous genetic, biochemical and clinical features may lead to the failure of the trial. For example, the drugs may be more effective in some ethnicities. Edaravone slowed the decline in the ALSFRS-R score and prolonged the survival time in Japanese (Writing Group, 2017) and American cohorts (Brooks et al., 2022), but a clinically relevant benefit was not observed in Europe. Patients with a shorter disease duration may have a better response to the drugs. For high-dose methylcobalamin, in a phase 2/3 study that included patients whose disease duration was less than 3 years, there was no significant difference in the ALSFRS-R score. However, the post hoc analyses showed that the drug slowed the progression of those within 12 months’ duration (Kaji et al., 2019). The criteria that selectively enroll patients with rapid or slow progression rates also increase the homogeneity of the population and thus have a better chance of efficacy being observed (Shefner et al., 2022). Therefore, using more specific inclusion criteria to recruit homogenous patients, especially those with a rapid progression rate, may increase the chance of finding effective drugs, although whether the results can be translated to a more general population remains a concern.

The observation duration of the trial may affect the results. Sometimes, the length of the observation period may not be long enough to observe significant results. In the phase 3 VALOR trial of tofersen, the ALSFRS-R score at the 28th week of its double-blinded trial did not differ significantly between the groups, but in the following open-label trial, earlier tofersen initiation was found to slow the decline of ALSFRS-R score, SVC and handheld dynamometry (Miller et al., 2022b). This suggested that the original designed observation duration was not long enough. Although the duration was determined on the limited data of a small population with rapidly progressing disease observed in its phase 1/2 study, due to the heterogeneity of disease, the progression rates of patients included in the VALOR may be not as fast as those observed in the phase 1/2 study. The baseline disease progression rates may not be sufficient for the identification of rapid and slow progression rates. Stringent disease and diagnosis may also influence the rate. However, the short observation period may be remedied by assessing the longer follow-up or use of demographic cofactors (Shefner et al., 2022).

The ALSFRS-R score is a common endpoint analyzed in clinical trials. It is a 12-item functional assessment evaluated from four domains. Although the analysis of the total score is widely used and has proven effective, some concerns have been raised regarding whether it will act poorly when the drug selectively improves one function (Shefner et al., 2022). Van Eijk et al. suggested analyzing on a subscale level to determine the benefit may avoid the pitfalls of the total score and enlarge the chance of observing effective outcomes (van Eijk et al., 2021). However, the different decline rates of individual subdomains are not addressed by this method (Shefner et al., 2022).

The analysis strategies of endpoints may affect the results. The ALSFRS-R score is most commonly analyzed as a continuous variable, using group statistics, in the form of slope of the rate of decline or mean change from baseline to the end (Shefner et al., 2022). Time to events such as death or tracheostomy are also used as the main endpoint. Some trials turned the continuous variable to events as the main endpoint. For example, CNM-Au8 used the percentage of patients with a 6-point decline in ALSFRS-R score as an endpoint. Although the percentage was decreased significantly (Meglio, 2022a), converting continuous variables to events may result in the loss of information. The responder analyses can help find subgroups that may respond more positively to the drugs. It is especially useful in a failed trial to isolate the possible responder and revise the inclusion criteria accordingly in the next clinical trial.

In conclusion, methylcobalamin, masitinib, AMX0035, CNM-Au8 and tofersen have shown the most potent therapeutic effects in clinical trials among the 53 new drugs. Population homogeneity, observation duration and analysis strategies are important factors that ensure the success of clinical trials.
